# Inflammation, fibrosis and E1 glycoprotein persistence in joint tissue of patients with post-Chikungunya chronic articular disease

**DOI:** 10.1590/0037-8682-0278-2023

**Published:** 2023-09-22

**Authors:** Maíra Sant Anna Genaro de Brito, Micheli Said de Marchi, Matheus Yung Perin, Isabelle da Silva Côsso, Renan Urt Mansur Bumlai, Waldemar Vaz da Silva, Adriana Yuki Mello Prado, Thais Campos Dias da Cruz, Eudes Thiago Pereira Avila, Amílcar Sabino Damazo, Renata Dezengrini Slhessarenko

**Affiliations:** 1 Universidade de Cuiabá, Faculdade de Medicina, Departamento de Clínica Médica, Cuiabá, MT, Brasil.; 2 Universidade Federal de Mato Grosso, Faculdade de Medicina, Programa de Pós-Graduação em Ciências da Saúde, Cuiabá, MT, Brasil.

**Keywords:** Chikungunya, Inflammatory, Cytokines

## Abstract

**Introduction:**

Chikungunya chronic joint disease causes debilitating arthralgia, significantly impacting the quality of life of affected individuals.

**Methods:**

In this study, patients underwent clinical follow-ups, joint biopsies, and pre-biopsy and 24 months post-biopsy serum dosage of cytokines.

**Results:**

All participants were female and had pain in 12 joints on average, with 41.17% exhibiting moderate disease activity. Histopathological analysis revealed collagen deposition. Indirect immunofluorescence detected the CHIKV glycoprotein E1 antigen, and an increase in cytokines.

**Conclusions:**

Persistent inflammation and ineffective antiviral immune responses leading to antigen persistence may contribute to chronic CHIKV arthritis.

*Chikungunya virus* (CHIKV) is an arthritogenic *Alphavirus* belonging to the*Martellivirales* order, *Togaviridae* family, transmitted to humans by arthropod vectors from *Aedes* genus^1^. CHIKV causes acute febrile disease accompanied by arthralgia and joint edema. Subsequently, many patients develop persistent or recurrent polyarthralgia in the following weeks, evolving to subacute and chronic debilitating articular disease^2^ ^,^ ^3^. 

Three hypotheses currently elucidate the development of chronic arthritis. These include local persistence of viral RNA directly linked to virus replication, antigen persistence hindering viral clearance and subsequently triggering chronic inflammation, and the continuation of nonprotective immune-mediated inflammation even after the resolution of CHIKV replication^4^ ^,^ ^5^ ^,^ ^6^. Histological studies enhance our understanding of viral pathogenesis by facilitating the identification of lesions and affected cell types. 

Elevated serum levels of cytokines, including IL-6, IL-12, IFN-α, IP-10, and MCP-1, were associated with an increased viral load, while IL-1β, TNF-α, IL-12, IL-10, IFN-γ, and IL-5 were linked to CHIKV joint disease progression in Italian patients^7^ ^,^ ^8^. Patients also exhibited persistent serum levels of IFN-α and IL-12^9^ and GM-CSF and IL-6, during the chronic phase^8^.

This longitudinal observational study sought to delineate the histological joint manifestations and serum cytokine levels in chronic CHIKV patients from the mid-southern region of Mato Grosso, following a significant outbreak of chikungunya fever from 2016 to 2018. 

We included 17 patients, all above 18 years, who attended the rheumatology medical clinic at the University of Cuiabá between July 2017 and September 2022. These patients were categorized based on the chikungunya fever diagnostic criteria^10^. Following their initial consultation and diagnosis of CHIKV articular disease, they underwent clinical and laboratory evaluations every three months if symptoms persisted. However, patients with concurrent systemic infections, metabolic diseases, or chronic autoimmune diseases were not included in the study.

Clinical data were prospectively collected from medical records and clinical evaluation forms utilized during each medical assessment. This information was employed to ascertain the severity of joint disease as per the Clinical Disease Activity Index (CDAI)^11^ ^,^ ^12^. 

Blood samples were collected to determine inflammatory markers and for cytokine dosage on the day the biopsy was performed (pre-biopsy) and 12-24 months after biopsy (post-biopsy).

Patients with chronic articular disease persisting for more than 12 months underwent joint biopsies. The selection of a specific joint was based on clinical manifestations and radiological findings. An orthopedist performed the procedure in an outpatient unit under aseptic conditions, beginning with local anesthesia using 1% lidocaine. 

The extracted fragment was dehydrated in an ethanol solution, cleared with xylol, and embedded in paraffin within 24 h of extraction. Thin sections (3 mm; HIRAX M60 Carl Zeiss; Germany) were deparaffinized, rehydrated, and subjected to hematoxylin-eosin staining to identify cellular structures and to special Masson trichrome staining, evidencing the muscular tissue and collagen fibers^13^. A single analyzer performed histological analyses on an AxioScope A1 microscope (Carl Zeiss, GR). 

Thin sections (3 µm) of joint biopsy tissue were prepared using HistoGrip^TM^ (Life Technologies, Maryland, USA). The sections were then deparaffinized and rehydrated. They were then incubated at 100 °C with 0.21% sodium citrate solution for 30 minutes, blocked with 3% H_2_O_2_ in 70% methanol for 60 minutes, and permeabilized with 0.4% tween 20 for 15 minutes. Subsequently, sections were blocked with 5% bovine serum albumin for 60 minutes and incubated overnight at 4 °C with an anti-CHIKV E1 glycoprotein mouse monoclonal antibody (anti-vCHIKV1; R&D Systems, USA; 1:200). Further, the tissue was incubated with goat anti-mouse IgG secondary antibody marked with ALEXAFLUOR 555 (Invitrogen, USA, 1:200) and nuclear DAPI marker (4’,6-diamidino-2-phenylindole) (Invitrogen™, USA) for 60 min in a dark chamber. The slides were mounted on Citiflour (DAKO, USA) and analyzed by a single analyzer using an AxioScope A1 microscope and the Axiovision software (Carl Zeiss, GR). Immunofluorescence was scored as follows: 0 = no signal; 1 = faintly marked cells; 2 = moderate; 3 = intense; 4 = very intense fluorescence detected^10^.

The second joint fragment was cut into small pieces with scalpel blades, eluted in RNase free water (0.4 mL) and subjected to total RNA extraction (0.2 mL) with Trizol LS (Thermo Fischer) and viral nucleic acid extraction (0.2 mL) including MS2 phage RNA (Sigma Aldrich) extraction control with MagMax viral pathogen kit (Applied Biossystems) following manufacturer’s instructions. 

Total RNA was subjected to an RT-PCR test targeting the CHIKV E1 region (305 bp). The viral nucleic acid was examined using the TaqMan Zika Virus Triplex Screening RT-qPCR Kit, which screens for Zika, Dengue, and Chikungunya viruses (Applied Biossystems). This testing was conducted on a Quantistudio 5 platform (Applied Biosystems). 

The inflammatory profile was assessed in the serum of 5 of 13 patients who continued to exhibit chronic disease on the day of the biopsy procedure and 11 of 13 patients 24 months post-biopsy. Venous blood was drawn into clot activator tubes, allowed to clot for 30 minutes, and then centrifuged for 15 minutes. 

The concentrations of cytokines and chemokines were ascertained using a Milliplex® Human Cytokine/Chemokine Magnetic Bead Panel (Millipore, USA). This panel is specifically designed to measure levels of TNF-α, IFN-α, INF-γ, IL-2, IL-4, IL-6, IL-8, IL-10, IL-12, IL-17A, IP-10, MCP-1, and GM-CSF. The measurements were conducted using dual-laser flow technology (Luminex® XMAP Technology, MAGPIX, Texas, EUA). 

The 17 patients, all women with an average age of 50 (+13.67) years, exhibited chronic CHIKV-articular disease for either 12 months (29.41%), 24 months (52.94%), or 36 months (17.64%) prior to undergoing joint biopsy. 

Clinically, patients presented a mean of 12 (1-28) painful and rigid articulations; 58.82% suffered arterial hypertension; 58.82% were using corticosteroids; 41.17% were taking non-steroidal anti-inflammatory drugs (NSAIDs); and none were using immunomodulators at the time of admission to the rheumatology service. 

All patients were negative for rheumatoid factor. Patients showed mean ferritin levels of 144.2 ng/mL (reference: 30 -300 ng/ml), mean C-reactive protein levels of 2.9 mg/L (reference: 3-10 mg/L) and a mean erythrocyte sedimentation rate of 30.5 mm/h (reference: < 50 years old, 20 mm/h; > 50 years old, 30 mm/h for women). 

On the day the biopsy was performed, all patients had normal inflammatory test results. With a mean of five painful joints, 47.05% had morning stiffness, and 5.88% were taking corticosteroids, 17.64% took NSAIDs, and 58.82% took immunomodulators. According to the CDAI, 17.64% of patients had high disease activity, 11.76% had moderate disease activity, 52.94% had low disease activity, and 17.64% were in remission. The mean value on CDAI at the time was 8.82 (Table 1).

**TABLE 1:** Clinical, histological and indirect immunofluorescence data of patients with post-Chikungunya chronic joint disease before and after joint biopsy.

Patient	Study inclusion	Age*	Duration of disease**	Comorbidities	CDAI		Therapy on biopsy day	Histopathology	IFI score***
					Study inclusion	Pre-biopsy			
01	04/2018	61	24	HBP	32	3	-	+++ diffuse inflammation in the fatty and in the connective tissue	0
02	12/2018	50	24	HBP	31	12	immunomodulator	++ inflammation, fat necrosis, +++ fibrosis****	2
03	12/2018	60	11	HBP/T2DM	22	24	-	+ inflammation, + fibrosis**** +++	2
04	05/2018	31	24	-	15	3	immunomodulator	+ inflammation +; ++ fibrosis	0
05	04/2018	62	7	HBP	10	3	NSAID+ immunomodulator	+ inflammation (synovitis); +++ fibrosis	3
06	03/2018	48	5	-	25	25	-	++ inflammation (synovitissynovitis); +++ fibrosis	3
07	08/2017	50	21	-	9	4	immunomodulator	+ inflammation (synovitis); +++ fibrosis	2
08	06/2018	44	30	-	35	5	NSAID	++ inflammation (synovitis); + fibrosis	1
09	02/2019	74	29	HBP	10	10	corticoid+ NSAID	+++ inflammation (histiocytes); + fibrosis	2
10	10/2018	56	33	-	11	0	immunomodulator	+++ inflammation (neutrophile infiltration); + fibrosis	2
11	02/2018	58	11	HBP/T2DM	12	6	immunomodulator	+ inflammation; +++ fibrosis	2
12	10/2018	52	33	HBP/T2DM	27	5	-	++ inflammation; +++ fibrosis	0
13	06/2018	57	7	HBP	5	2	immunomodulator	+ inflammation in the fat cells; +++ fibrosis	3
14	03/2018	50	35	-	13	12	-	+ inflammation; ++ fibrosis	
15	12/2018	38	35	HBP/T2DM	12	25	immunomodulator	+ inflammation; +++ fibrosis	2
16	06/2019	24	29	-	21	6	immunomodulator	+ inflammation + (synovitis); +++ fibrosis	1
17	10/2018	36	32	HBP/T2DM	30	8	immunomodulator	++ inflammation (synovitis); +++ fibrosis	2

*In years, when included in the study. ** Duration of disease, in months, on the day biopsy was performed. *** Chikungunya E1 antigen detection by indirect immunofluorescence (IFI). **** fibrosis was defined by collagen deposition in the joint tissue. **CDAI:** clinical disease activity index; **HBP:** high blood pressure - hypertension; **T2DM:** diabetes mellitus type 2; **NSAID:** non-steroidal anti-inflammatory drugs.

In the last clinical evaluation in October 2022 (24 months after biopsy), 12 out of 17 patients still had a mean of eight tender joints, 64.7% presented persisting matinal stiffness, 11.76% were using systemic corticosteroids, while 17.64% were taking NSAIDs. Furthermore, 41.17% of the patients were undergoing immunomodulatory therapy with methotrexate, hydroxychloroquine, sulfasalazine, or a combination of these drugs^11^. The CDAI showed that 41.17% patients presented low disease activity (mean: 12.52), 23.52% had high disease activity, 17.64% moderate disease activity, and 17.64% were in remission (Table 1). Histopathological findings in the joint fragments included in fibrosis (10 of 17) (Figure 1), associated with intense (3 of 17) or discrete (14 of 17) inflammation; one patient also had a periarticular inflammatory infiltrate (Figure 1). Four patients showed predominant synovitis, one had synovitis with the presence of histiocytes, and one patient presented fat necrosis (Figure 1). CHIKV E1 antigen was detected in 14 of 17 joint tissues, 57.14% with moderate staining (Figure 1). 

**FIGURE 1:**
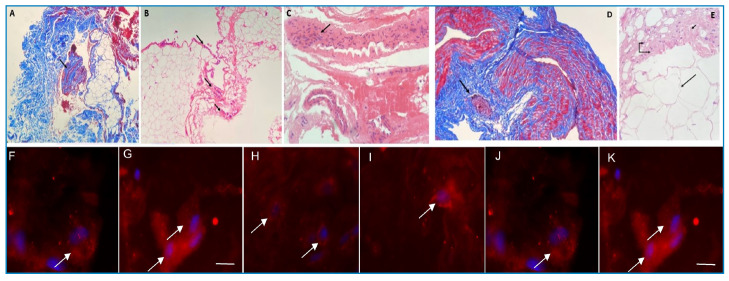
Histological analysis (40x magnification) and immunofluorescence for chikungunya E1 antigens (Bars = 5uM) of joint tissue of patients with post-CHIKV chronic disease. **(A)** Mild synovitis (arrow) and extensive collagen deposition (blue) by Masson´s special trichrome staining. **(B)** Diffuse inflammatory process (arrows) over connective tissue by hematoxylin-eosin staining. **(C)** Synovitis in joint tissue (arrow) by hematoxylin-eosin staining. **(D)** Intense inflammatory process (arrow) and presence of collagen deposition (blue) in joint tissue by Masson's special trichrome staining. **(E)** Fat necrosis (long arrow), moderate inflammatory process (double arrow) and collagen deposition (short arrow) in joint tissue by hematoxylin-eosin staining. **(F-K)** arrows indicate immunostained cells for CHIKV E1 antigen. Nuclei of cells are stained with DAPI (blue).

All 17 fragments tested negative for viral RNA, as determined by both RT-PCR and RT-qPCR.

Table 2 displays the CDAI scores and average concentrations of various cytokines/chemokines measured in patient serum samples before and after biopsy. Notably, the levels of TNF-α, MCP-1, IP-10, IL-17, IL-4, and IL-2 were elevated in all patient samples, both pre- and post-biopsy. 

**TABLE 2:** Clinical disease activity index (CDAI) and serum levels of cytokines and chemokines pre- and post-biopsy procedure in patients with chronic arthralgic disease induced by Chikungunya virus.

Patient	Age (years)	Duration of disease (months)*	CDAI		CDAI			Cytokines/chemokines (pg/mL)													
			Study inclusion	Pre-biopsy	12 months post-biopsy	24 months post-biopsy	Time**	IL-7	GM-CSF	IFN-α	TNF-α	IL-6	IL-8	IL-12p70	IL-2	IL-4	IFN-γ	IL-17a	IL-10	IP-10	MCP-1
01	61	24	32	3	0	10	Pre-	76	29	31	71	55	116	37	40.5	162	65	34	31	4,921	660.5
							Post	158	23	20	26	24	187.5	22	26	74	52	23	27	652	975
02	50	24	31	12	19	14	Post	72	20	19	27	34	119.5	20	26	51	40	21	18.5	2,187	2,968
03	60	11	22	24	5	30	Post	54	16	14	20	33	365.5	19	21	57	31	20	32	5,172	5,784
04	31	24	15	3	0	8	Post	60	18	14	42	37	122.5	17	22	28	29	17	24	2,264	3,640
05	62	7	10	3	5	4	Pre-	68	20	21	35	32	99.5	22	25	70	46	20	28	3,499	996
06	48	5	25	25	0	0	Post	36	16	10	23	41.5	394	13	18	33	43	16	19	1,301.5	5,225
07	50	21	9	4	5	3	Pré-	188	19	19	25	24	199	23	30	82	54.5	22	29	803	1383
08	44	30	35	5	4	33	ND	-	-	-	-	-	-	-	-	-	-	-	-	-	-
09	74	29	10	10	0	0	ND	-	-	-	-	-	-	-	-	-	-	-	-	-	-
10	56	33	11	0	3	25	Post	151	23	17	30.5	32	82.5	18	29	42	38	21	21	2,758	2,466
11	58	11	12	6	11	15	Pre-	42	20	19	27	32	169	19	28	53	70	20	33	6,252.5	3,215.5
							Post	60	17	19	24.5	34	198	20	24	17	28	28	29	505	4,146.5
12	52	33	27	5	0	9	Post	62	22	21	37	29.5	138	23.5	25	40	34	25	27	1619	2456
13	57	7	5	2	2	8	Pre-	24	14	13	14	31	164	16	18	18	24	15	21	1,508	4,767
							Post	51.5	20	20	26	24	122.5	23	25	32	32.5	20	26	1,678	2,492.5
14	50	35	13	12	0	0	Post	53	20	15	31	59.5	74	20	23	44	23	18	21	1,157	1,482
15	38	35	12	25	10	20	ND	-	-	-	-	-	-	-	-	-	-	-	-	-	-
16	24	29	21	6	0	7	Post	69	27	32	51	46	115	1,349	40	87	55	34	47	9,156	2,136
17	36	32	30	8	26	27	ND	-	-	-	-	-	-	-	-	-	-	-	-	-	-

*When biopsy was collected. **Cytokines/chemokines dosed pre-biopsy (pre) and 24 months after biopsy in the last clinical evaluation (post). **ND:** not determined.

Only one patient’s post-biopsy sample showed increased levels of IL-10, which is an anti-inflammatory cytokine, and a second patient had post-biopsy increases in the levels of chemokine IL8.. Two patients showed increased levels of IL-12 before the biopsy. One of these patients had normal IL-12 levels after the biopsy. IFN-γ levels were increased in the serum of six patients, three pre-biopsy and three post-biopsy. One patient who had elevated IFN-γ levels prior to the biopsy had normal levels of this cytokine after the biopsy. 

IFN-α was increased in the serum of four patients, only one patient had increased IFN-α pre- and post-biopsy, the same patient showed increased levels of IL-12 only before the biopsy. IL-6 and GM-CSF levels were within reference values in all patients. 

Over 50% of CHIKV chronic patients attending to our service experienced ≥24 months of post-CHIKV chronic inflammatory joint disease. Bouquillard et al. reported 83.1% joint pain persistence over a 32-month follow-up but couldn't detect viral RNA using RT-PCR in some cases^14^. Similarly, we also failed to detect viral RNA in joint fragments using two molecular protocols. During the outpatient follow-up, we observed a gradual decrease in the number of affected joints, by the analog pain score, and the CDAI values. This was accompanied by the maintenance of C-reactive protein, ferritin, and erythrocyte sedimentation rate within normal limits. Additionally, we noted a decrease in the levels of inflammatory cytokines and chemokines, which corresponded with the patient’s clinical improvement between the pre- and post-biopsy measurements (Table 2). 

The collagen deposition observed in most joint samples in this study supports the notion that long-term intra-articular changes, induced by sustained inflammation, result in fibrosis. However, in our study, none of the patients tested positive for autoantibodies. 

The epidemiological profile of these patients, predominantly middle-aged women with comorbidities, supports immunopathological studies indicating a reduction in viral clearance due to immune response dysregulation. This could partially account for the persistence of severe articular disease induced by CHIKV and the absence of E1 antigen clearance in the joints within this population^4^. 

The overrepresentation of female patients in our study could be attributed to their health-seeking behavior and the presence of risk factors for prolonged joint pain specific to this gender^15^. However, the study lacks supplementary data to elucidate the pathophysiological basis for the observed increase in chronic cases among females. 

The chronic phase of the disease may be perpetuated by immune activation due to the persistent presence of viral RNA and E1 antigen in the synovial and muscle tissues. This results in the continuous production of inflammatory cytokines and chemokines^16^ **.** Our study was unable to demonstrate whether the persistence of CHIKV RNA is due to viral replication or ineffective proinflammatory responses. While we detected persistent E1-antigen in the joint tissues, we were unable to detect viral RNA. 

Chronic articular disease caused by the Chikungunya virus has been linked to elevated serum levels of MCP-1, IL-6, IL-10, IFN-α, and GM-CSF in several cohort studies^4^. In certain chronic patients, an increase in serum levels of IFN-α and IL-12 has been correlated with the persistence of the inflammatory process and disease progression^9^. Our patients exhibited significantly elevated serum levels of TNF-α, MCP-1, IP-10, IL-17, IL-4, and IL-2. The normal levels of GM-CSF and IL-6 observed in our study align with findings in chronic CHIKV cases, as increased circulating levels of this cytokine are typically present in acutely infected patients, with levels decreasing in individuals who have recovered^7^. 

All our patients had increased levels of IFN-induced protein IP-10, with six of them also exhibiting heightened IFN-γ responses. However, IL-4 and IL-17, typically associated with Th2 and Th17 subtypes, were increased in the serum of all dosed patients.

The introduction of immunomodulatory therapy during the patient’s follow-up resulted in a decreased requirement for symptomatic drugs, including corticosteroids and NSAIDs. Our data revealed that, following a 30-month period, 43% of patients recovered from the disease, 35% exhibited mild disease activity, and 22% continued to display moderate disease activity. 

The study had limitations due to a small number of patients undergoing joint biopsy and long-term evaluations. However, it revealed enduring inflammatory responses, intra-articular fibrosis signs, and viral E1 antigen persistence in patients with post-CHIKV articular complaints for ≥12 months. Elevated pro-inflammatory cytokine serum levels persisted even as CDAI indicated reduced pain complaints, over at least 29 months of follow-up.

## ETHICS

Procedures involving human subjects and their samples received prior approval from the Health Ethics Council, Medicine School, Propeq/UFMT (CAEE 77593417.7.0000.8124 approval 2.658.648). This study adhered to the National Health Council resolution 196/96. All patients were thoroughly briefed about the study procedures and subsequently provided their informed consent.
